# Inhibitory activity of Indian spice plant *Cinnamomum zeylanicum *extracts against *Alternaria solani *and *Curvularia lunata*, the pathogenic dematiaceous moulds

**DOI:** 10.1186/1476-0711-8-9

**Published:** 2009-03-07

**Authors:** Ajay K Mishra, Amita Mishra, HK Kehri, Bechan Sharma, Abhay K Pandey

**Affiliations:** 1 Department of Biochemistry, University of Allahabad, Allahabad, 211002, India; 2 Department of Botany, University of Allahabad, Allahabad, 211002, India

## Abstract

**Background:**

Dematiaceous moulds are pathogenic microorganisms and act as etiological agents of mycoses with different degrees of severity in humans and animals. These moulds also cause loss of food crops and storage food products. The information regarding antimicrobial efficacy of the plant preparations on these moulds is scanty. The present study reveals phytochemical characterization and the effect of bark and leaf extracts of Indian spice plant, *Cinnamomum zeylanicum *(Cz), against the growth of two species of dematiaceous moulds, *Alternaria solani *and *Curvularia lunata*.

**Methods:**

Cz bark and leaf samples were sequentially extracted in different solvents using Soxhlet apparatus. Phytochemical analyses of extracts were done as per standard protocols. The antifungal bioassay of extracts was done by hanging drop technique. The inhibition of fungal spore germination was monitored under influence of three different concentrations of extracts.

**Results:**

The lowest test concentration (50 μg/ml) of extracts of Cz bark prepared into acetone and that of Cz leaf into petroleum ether and ethanol exhibited complete inhibition (100%) of spore germination in both the moulds. At 100 μg/ml concentration all the extracts showed about 50 to 100% inhibition. However, the treatment of the spores of the two fungal species with highest concentration (500 μg/ml) of bark and leaf extracts in all the solvents showed 100% fungicidal activity as it completely arrested the germination of spores. Relatively lower activity of aqueous extracts at 50 and 100 μg/ml concentrations suggests that the antifungal ingredients present in Cz bark and leaf are more soluble in organic solvents than water.

**Conclusion:**

The results demonstrated that the Cz bark and leaves contain certain fungicidal constituents exhibiting potential antimould activity against *A. solani *and *C. lunata*.

## Background

The dematiaceous moulds are characterized by presenting melaninogenic pigmentation in their cell walls. It is the main characteristic of the dark moulds group. Some authors have reported dematiaceous moulds as opportunistic pathogens generally with low pathogenicity which could get into the human or animal body by repeated traumatic inoculation [1,2]. The two dematiaceous moulds such as *Alternaria *spp. *and Curvularia *spp. are known potential etiological agents of various mycoses with clinical forms ranging from localized superficial infections of the stratum corneum (Tinea nigra) to subcutaneous cysts (phaeomycotic cyst) to invasion of the brain [3]. The anti-dematiaceous moulds therapy has been carried out by the use of some antibiotics such as amphotericin B, 5-fluorocitosin and itraconazole [4,5]. *Curvularia *has been reported to cause infections in both humans and animals [6-9].

Dematiaceous moulds are also plant pathogens and cause diseases of cultivated crops posing serious threat to increased agricultural production. The protection of plants from pathogens has always been of prime concern in order to get better yield. *Alternaria solani *causes a destructive foliar disease in potato and tomato plants (family solanaceae) known as early blight. The early symptoms are in the form of small, yellowish brown spots on the leaves, which enlarge in size and become round to form black concentric rings, often killing most of the plant in the long run. Seeds infected with this pathogen may even damp off during germination. Another damatiaceous mould is *Curvularia*. Most species of this fungus are facultative pathogens of plants and cereals in tropical or subtropical areas, while the remaining is found in temperate zones. In addition to many other crops, *Curvularia *grows on paddy [10] causing leaf spots, blights, grain deformation, grain discolouration and even root rot.

The phytochemicals acting as antimicrobials represent a vast untapped source for medicines and hence have enormous therapeutic potential. They are effective in the treatment of infections while mitigating many of the side effects associated with synthetic antimicrobial and antibacterial agents [11,12]. The pharmacologically active plants have supplied over 25% of prescription drugs used in human medicine. In addition, as there is always a need to develop new biofungicides from natural plant sources; some plant products have been shown to act as natural pesticides [13].

There are some reports on antimicrobial activity of *Cinnamomum zeylanicum *(Cz) against Gram positive and Gram negative bacteria, viruses, moulds and yeasts. The results have ranged according to the microorganism and assayed product (essential oil, extracts, decoct, plant powder). Phytochemicals are small organic biomolecules generally hydrophobic and designated as naturally occurring antibiotics [14-16]. Antifungal property of phytochemicals could involve cytosolic hyperacidity, breakage of electrons transport chain, H^+^-ATPase inhibition, channels inhibition, intracellular and extracellular enzymes synthesis inhibition [17].

Though the antimicrobial activity of certain plant products such as extracts, essential oils and phytochemicals has been reported [18-20], the review of literature indicates that no systematic study has been conducted regarding application of phytochemicals extracted from Indian spice plant, Cz, as antifungal agents. In the present communication, an endeavour has been made to determine phytochemical profiles of Cz bark as well as leaf extracts and to evaluate the antimould activity of these extracts against the germination of spores of two dematiaceous moulds such as *Alternaria solani *and *Curvularia lunata*.

## Methods

### Plant Material

The bark and leaves of Cz were collected from Forest Research Institute, Dehradun in October/November 2006. Freshly collected plant parts were shade-dried at room temperature for 10–15 days. Dried bark and leaf samples were separately crushed and ground into fine powder with mortar and pestle.

### Preparation of extracts

Powdered plant materials were sequentially extracted with different solvents in a Soxhlet apparatus for 8 h according to the method described elsewhere [21]. The solvents used for extraction included petroleum ether (PE), benzene (BZ), chloroform (CH), ethyl acetate (EA), acetone (AC), ethanol (ET) and water (AQ). The respective extracts were filtered and dried under reduced pressure using rotary evaporator to yield solid/semisolid residues. The residues were lyophilized to get dry solid mass.

### Phytochemical analysis

Qualitative phytochemical analysis of Cz bark and leaf extracts was done as follows:

### Tannins

20 mg extract was dissolved in 2 ml distilled water and filtered. 2 ml FeCl_3 _was added to the filtrate, blue-black precipitate indicated the presence of tannins [22].

### Alkaloids

20 mg extract was dissolved in 2 ml distilled water and filtered. To the filtrate, 2–4 drops of 1% HCl was added and steam was passed through it. To the 1 ml of this solution 6 drops of Wagner's reagent was added. Brownish-red precipitate indicated the presence of alkaloids [22].

### Saponins

To 0.5 ml of the filtrate obtained in alkaloids test 5 ml distilled water was added. Frothing persistence indicated the presence of saponins [22].

### Flavonoids

20 mg extract was dissolved in 10 ml ethanol and filtered. 0.5 ml conc. HCl and magnesium ribbon were added to 2 ml filtrate. Development of pink-tomato red color indicated the presence of flavonoids [22].

### Terpenoids

Salkovski test was performed using a small amount of extract solution. To this solution 5 drops of conc. H_2_SO_4 _and 1 ml Chloroform were added. Change of yellow colour into red indicated the presence of terpenoids [23].

### Phenols/polyphenols

A small amount of material was extracted in ethanol and evaporated to dryness. Residue was dissolved in distilled water and 0.5 ml Folin-ciocalteau reagent was added followed by 2 ml 20% Na_2_CO_3 _solution. Development of bluish colour indicated the presence of phenols [24].

### Test moulds

*A. solani *and *C. lunata *were isolated from soil on potato dextrose agar (PDA) plates. These dematiaceous moulds were grown and maintained on PDA slants at 28 ± 1°C. Following incubation for five days, the cultures were either utilized for test or stored at 4 ± 1°C for further use. The organisms were subcultured once in every fifteen days and the purity of the cultures was checked regularly under microscope.

### Bioactivity testing

For the study of antimould activity, several concentrations of extracts (50, 100 and 500 μg/ml) were prepared in 5% DMSO. Bioactivity of the extracts was determined by counting spore (conidia) germination at various concentrations of extracts after 5 h of incubation through hanging drop technique [25]. Briefly, the spores were isolated from PDA slants and transferred in 1 ml of each extract prepared in 5% DMSO. 50 μl extract containing spores was transferred at the centre of glass coverslip and number of spores was counted with the help of microscope. The coverslips containing spore suspension were placed on different depression slides containing a drop of water. The slides along with hanging spore suspension were incubated for 5 h at 28 ± 1°C. The spores were further counted under microscope to evaluate the effect of extracts on germination. Blank test showed that 5% (v/v) DMSO used in the preparations of the test solutions does not affect the spore germination of test organisms. All experiments were carried out in triplicate and data were expressed as mean ± SEM. The bar diagrams were prepared using Graphpad Prism software.

## Results

### Phytochemical Analysis

Results of phytochemical analysis of Cz bark and leaf extracts are given in Table 1 and Table 2, respectively. Extracts were tested for the presence of phenols/polyphenols, flavonoids, terpenoids, tannins, alkaloids and saponins or the combinations thereof. Phenols and terpenoids were invariably present in all the bark extracts. However, only phenols were present in all the solvent fractions of leaf. The analysis of different bark and leaf extracts also showed presence of combinations of other phytochemical constituents.

**Table 1:** Phytochemial analysis *C. zeylanicum *bark extracts

	Extracts						
	
Phytochemicals	PE	BZ	CH	EA	AC	ET	AQ
Phenol/polyphenols	+	+	+	nt	+	+	+

Flavonoids	-	+	+	nt	+	+	-

Terpenoids	+	+	+	nt	+	+	+

Tannins	-	-	+	nt	+	+	+

Alkaloids	+	-	-	nt	-	+	+

Saponins	+	-	-	nt	-	+	nt

Phytochemical analysis of different extracts of *C. zeylanicum *bark was done as shown in Methods section. PE = petroleum ether; BZ = benzene; CH = chloroform; EA = ethyl acetate; AC = acetone; ET = ethanol; AQ = water; nt = not tested; (+) present/detected; (-) not detected.

**Table 2:** Phytochemial analysis of *C. zeylanicum *leaf extracts

	Extracts						
	
Phytochemicals	PE	BZ	CH	EA	AC	ET	AQ
Phenol/polyphenols	+	+	+	+	+	+	+

Flavonoids	-	+	+	+	+	+	-

Terpenoids	+	-	-	-	+	+	+

Tannins	-	-	-	-	+	+	+

Alkaloids	+	-	-	+	-	+	+

Saponins	+	-	-	-	-	+	nt

Phytochemical analysis of different extracts of *C. zeylanicum *leaves was done as shown in Methods section. PE = petroleum ether; BZ = benzene; CH = chloroform; EA = ethyl acetate; AC = acetone; ET = ethanol; AQ = water; nt = not tested; (+) present/detected; (-) not detected

### Inhibition of spore germination by different plant extracts

**Figure 1:**
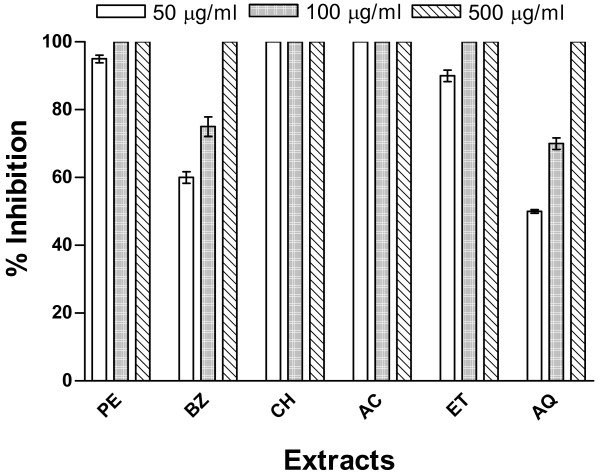
**Inhibition of germination of spores of *A. solani *by *C. zeylanicum *(Cz) bark extracts**. The Cz bark extracts were prepared in (1) petroleum ether (PE), (2) benzene (BZ), (3) chloroform (CH), (4) acetone (AC), (5) ethanol (ET), and (6) water (AQ) as described in Methods section. Three concentrations (50, 100 and 500 μg/ml) have been used to evaluate the inhibitory activity of extracts against germination of spores of *A. solani *under experimental conditions as mentioned in experimental section.

**Figure 2:**
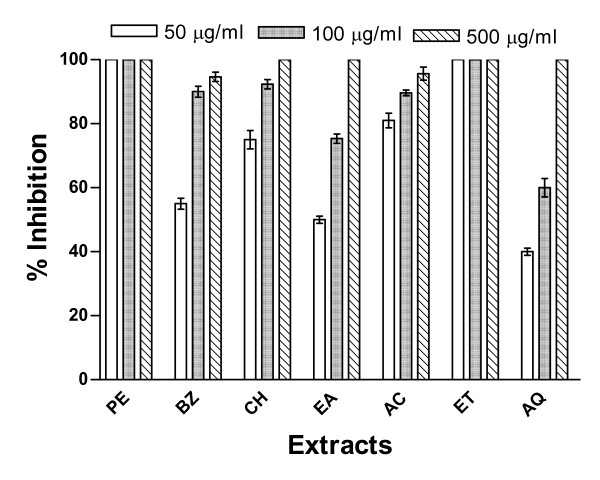
**Inhibition of germination of spores of *A. solani *by *C. zeylanicum *(Cz) leaves extracts**. The Cz leaves extracts were prepared in (1) PE, (2) BZ, (3) CH, (4) ethyl acetate (EA), (5) AC, (6) ET, and (7) AQ as described in Methods section. Other conditions were same as described in Fig. 1.

**Figure 3:**
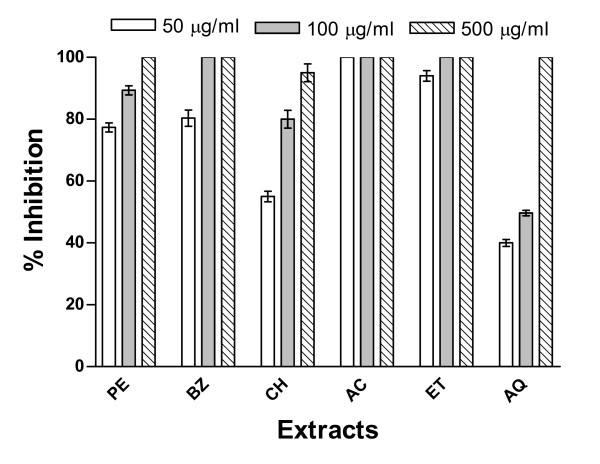
**Inhibition of germination of spores of *C. lunata *by *C. zeylanicum *(Cz) bark extracts**. The Cz bark extracts were prepared in (1) PE, (2) BZ, (3) CH, (4) AC, (5) ET, and (6) AQ as described in Methods section. Three concentrations (50, 100 and 500 μg/ml) have been used to evaluate the inhibitory activity of extracts against germination of spores of *C. lunata *under experimental conditions as mentioned in experimental section.

**Figure 4:**
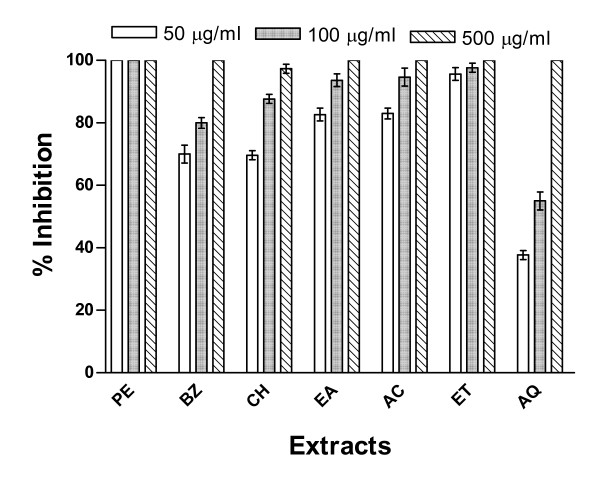
**Inhibition of germination of spores of *C. lunata *by *C. zeylanicum *(Cz) leaves extracts**. The Cz leaves extracts were prepared in (1) PE, (2) BZ, (3) CH, (4) EA, (5) AC, (6) ET, and (7) AQ as described in Methods section. Other conditions were same as described in Fig. 3.

The inhibition of spore germination of two different species of moulds namely *A. solani *and *C. lunata *by extracts of Cz bark and leaves was examined at three different concentrations (50, 100 and 500 μg/ml). Both of the mould species were found sensitive to the treatment with these extracts. Results of antimould activity of bark and leaf extracts on *A. solani *are shown in Figures 1 and 2 while their effects on *C. lunata *are depicted in Figures 3 and 4.

### Effect of Cz bark extracts on the germination of spores from *A. solani *and *C. lunata*

The Cz bark extracts prepared in chloroform and acetone were found to be most active against germination of spores of *A. solani *as their lowest dose (50 μg/ml) was sufficient to prevent 100% germination (Fig. 1), while in case of *C. lunata *only acetone extract of bark could exert complete inhibitory effect (Fig. 3). The results demonstrated in Figure 1 reflected that at 50 μg/ml concentration, the extracts prepared into petroleum ether and ethanol exhibited about 90–95% activity against spores' germination of *A. solani*, whereas those prepared in water and benzene could inhibit this activity by about 50 and 60%, respectively. The inhibitory potential at all the three concentrations tested with extracts prepared into petroleum ether and chloroform were similar to those of ethanol and acetone, respectively. The bark extracts into petroleum ether, chloroform, acetone and ethanol completely arrested the spores germination of *A. solani*, whereas aqueous and benzene preparations could inhibit this activity by about 70 and 76%, respectively, at 100 μg/ml concentration. The highest concentration (500 μg/ml) of all the extracts tested caused complete (100%) inhibition of germination of *A. solani *spores. The inhibitory effect of these extracts from bark was found to be maximum with chloroform or acetone extract and minimum with aqueous preparation.

These extracts when tested against the germination of *C. lunata *spores displayed a different pattern (Fig. 3). The extract in acetone completely inhibited the germination of spores at minimum concentration tested (50 μg/ml). The ethanolic extract was almost equally active as it could show about 95% inhibition at this concentration. However, other extracts at this concentration could display inhibitory effect up to 35–80% only. The bark extracts at higher concentration (100 μg/ml) could exhibit 100% inhibition in the spores' germination of *C. lunata *only when prepared in benzene, acetone and ethanol. The ethanol extract showed almost similar inhibitory pattern with that of acetone extract at all the three concentrations tested. The treatment of *C. lunata *spores with highest concentration (500 μg/ml) of all the test extracts resulted in almost complete inhibition of germination. The Cz extract in acetone was most active against *C. lunata *while the aqueous extract proved to be relatively least active (Fig. 3).

### Effect of Cz leaf extracts on the germination of spores from *A. solani *and *C. lunata*

Since the data obtained from above experiments conducted with the extracts of bark demonstrated significant fungicidal potential, the attempts were made to evaluate the antifungal property of the extracts of leaves prepared in the aforesaid solvents. The results obtained after treatment of *A. solani *spores with the extracts of leaves showed a different trend in inhibitory activity. The data presented in Figure 2 indicated that the lowest concentration (50 μg/ml) of leaves extracts prepared in ethanol and petroleum ether was most active and caused 100% inhibition followed by the acetone, chloroform benzene and aqueous extracts. However, all other extracts of leaves were able to inhibit completely the spores' germination of *A. solani *only at the highest concentration (500 μg/ml). At lower concentrations (50 and 100 μg/ml) all the extracts (excepting those prepared in petroleum ether and ethanol) registered only 40–80% and 60–95% inhibition, respectively. The aqueous extract from the leaves exhibited relatively lesser inhibitory activity at lower concentrations (Fig. 2). These extracts when tested against the germination of *C. lunata *spores, the lowest concentration of leaves extract prepared in petroleum ether could show complete (100%) inhibition followed by the extracts prepared in other solvents (Fig. 4). The inhibitory activity pattern of the extracts prepared in benzene and ethyl acetate were similar to those of chloroform and acetone, respectively, at all the three concentrations tested. The ethanol extract exhibited close proximity in the inhibitory efficacy with that of petroleum ether extract at all the concentrations tested (Fig. 4).

The complete inhibition (100%) of spore germination in both these moulds was observed with acetone extract of Cz bark (Figs. 1 and 3) as well as with petroleum ether and ethanol extracts of Cz leaf (Figs. 2 and 4) at the lowest concentration (50 μg/ml). At 100 μg/ml concentration all the extracts exhibited about 100% inhibition of spore germination of these two mould species. However, complete inhibition (100%) of spore germination was observed at a concentration of 500 μg/ml for all extracts from both the bark and leaf. About 40–50% reduction in spore germination was observed for these two moulds at the lowest test concentration (50 μg/ml) of aqueous extract.

## Discussion

Antifungal susceptibility testing remains an area of intense interest. Susceptibility testing can be used for drug discovery and epidemiology. Number of reports is available showing efficacy of Cz essential oils as antimicrobial agents [16,20]. The oil extracted from Cz bark and leaves have been reported to possess fungicidal activity against fungi responsible for causing crown rot disease of banana. The major constituent possessing antifungal activity in Cz bark and leaf oils were found to be cinnamaldehyde and eugenol, respectively. In addition other compounds having fungicidal property have also been reported to be present in bark and leaves [26-28]. However, the data regarding use of Cz extracts as antifungal agents are scanty.

Despite serious environmental implications associated with the excessive use, chemical fungicides still remain the first line of defense against fungal pathogens. Moreover, these fungicides when ingested by human beings and animals through food and water cause various ailments in the body. Search of natural fungicidal principles from the plant sources would definitely be a better alternative to these hazardous chemicals. Our study has indicated the antimould potential of plant extracts, as the Cz bark and leaf extracts displayed complete inhibitory effect on spore germination of aforesaid two dematiaceous moulds.

The organic and aqueous extracts of Cz bark and leaves studied in the current work showed marked antimould activities against *A. solani *and *C. lunata *responsible for causing diseases in animals and plants as well as spoilage of stored food products. These organisms pose important public health and economic concerns for human society. However, the extracts differ significantly in their activity against the above moulds. The differences observed in the bioactivity assays suggest the susceptibility of these moulds to various secondary metabolites present in this endemic plant. The composition of these secondary metabolites in turn varies from species to species and also on climatic conditions and the physiological state of developments of the plants [29].

The relative antimould activity of Cz extracts may not be easily correlated with any individual component but with a mixture of compounds present in these extracts. There are reports showing that alkaloids and flavonoids are the responsible compounds for the antifungal activities in higher plants [30]. Moreover, secondary metabolites such as tannins and other compounds of phenolic nature are also classified as active antimicrobial compounds. Phenols, the aromatic compounds with hydroxyl groups are widespread in plant kingdom. They occur in all parts of plants. Phenols are said to offer resistance to diseases and pests in plants. Grains containing high amount of polyphenols are resistant to bird attack [24]. Interestingly, phytochemical screening of the current investigation has revealed that extracts from both the plant parts possess at least three to four of the following classes of secondary metabolites: phenols, flavonoids, terpenoids, tannins, alkaloids and saponins. Therefore, the presence of these phytochemicals could to some extent justify the observed antifungal activities in the current study. These results are in agreement with many studies realized on other plant species belonging to the euphorbiaceae [29] and asteraceae [31] attributing antimicrobial activities to the presence of secondary metabolites.

## Conclusion

Our results have established the intense antimould potential of *C. zeylanicum *extracts against *A. solani *and *C. lunata*. It could be regarded as promising alternative antimicrobial preparation to be inserted in pharmaceutical formulations used to treat mycoses of different clinical severities and the plant diseases, particularly, those caused by dematiaceous moulds. The aforesaid Indian spice plant contains phytochemicals to be developed as prospective antifungal agents.

## Abbreviations

Cz: *Cinnamomum zeylanicum*; *A. solani*: *Alternaria solani*; *C. lunata*: *Curvularia lunata*; PE: petroleum ether; BZ: benzene; CH: chloroform; EA: ethyl acetate; AC: acetone; ET: ethanol; AQ: water.

## Competing interests

The authors declare that they have no competing interests.

## Authors' contributions

AKP and BS participated in the research design, analysis of the data and drafting the manuscript. AKM and HKK carried out the antimicrobial activity tests. AKM and AM collected the materials for the extract preparation and conducted related experiments. All authors have read and approved the final manuscript.
